# Cocaine-induced acute myocardial infarction: angiographic features and outcomes

**DOI:** 10.1007/s00392-025-02677-6

**Published:** 2025-05-27

**Authors:** Michael Würdinger, Davide Di Vece, Victor Schweiger, Iva Koleva, Barbara E. Stähli, Jelena-Rima Ghadri, Erik W. Holy, Christian Templin

**Affiliations:** 1 Department of Cardiology, University Heart CenterUniversity Hospital Zurich, and University of Zurich, Zurich, Switzerland; 2 Internal Medicine B, University Medicine Greifswald, Ferdinand-Sauerbruch-Straße, 17475 Greifswald, Germany; 3 First Clinic of Internal Medicine, Department of Internal Medicine, University of Genoa, 6 Viale Benedetto XV, 16132 Genoa, Italy; 4 Center for Molecular Cardiology, Schlieren Campus, University of Zurich, Zurich, Switzerland; 5 Department of Angiology, University Hospital Zurich, and University of Zurich, Zurich, Switzerland; 6 Private Hospital Bethanien, SwissCardioVascular Clinic (SwissCVC), Zurich, Switzerland

**Keywords:** Cocaine, Myocardial infarction, Acute coronary syndrome, Spontaneous coronary artery dissection, Coronary microvascular dysfunction, Takotsubo

## Abstract

**Background:**

Cocaine is a global health burden and the cause of a significant number of emergency department consultations. Its association with acute myocardial infarction (AMI) is known, however, data are still rare. The aim of this study was to define causative pathologies behind cocaine-induced AMI (CI-AMI) and to analyze their clinical features.

**Methods:**

Patients with the diagnosis of CI-AMI were retrospectively identified at the University Hospital Zurich between 1997 and 2023. The angiograms were reviewed to confirm the diagnosis. Coronary microvascular dysfunction (CMD) was separately evaluated by an angiography-based analysis (AngioPlus Core, Microport Medical Co.). The primary endpoint was rates of major adverse cardiovascular events (MACE) at 30 days, 1 year, and 2 years.

**Results:**

Forty-five cases of CI-AMI were identified. Twelve patients (27%) were diagnosed with plaque rupture and intraluminal thrombus, eight (18%) with coronary artery disease (CAD) without thrombus formation, eight (18%) with spontaneous coronary artery dissection, six (13%) with CMD, four (9%) with coronary vasospasm, and four patients (9%) with Takotsubo syndrome. The cause of CI-AMI remained unclear in three patients (6%). No clinically useful predictors of CAD were identified. 91% of patients had values associated with CMD during angiography-based analysis, independently from the etiology of CI-AMI. 49% of cases were treated by revascularization, and the number of MACE was high (16%, 28%, and 34% at 30 days, 1 year, and 2 years).

**Conclusions:**

CI-AMI is a rare, but important cause of acute coronary syndromes (ACS). CAD represents the most frequent etiology of AMI, but there is a broad range of other entities. Patients suffer from a significant number of adverse events.

**Graphical abstract:**

**Figure:**
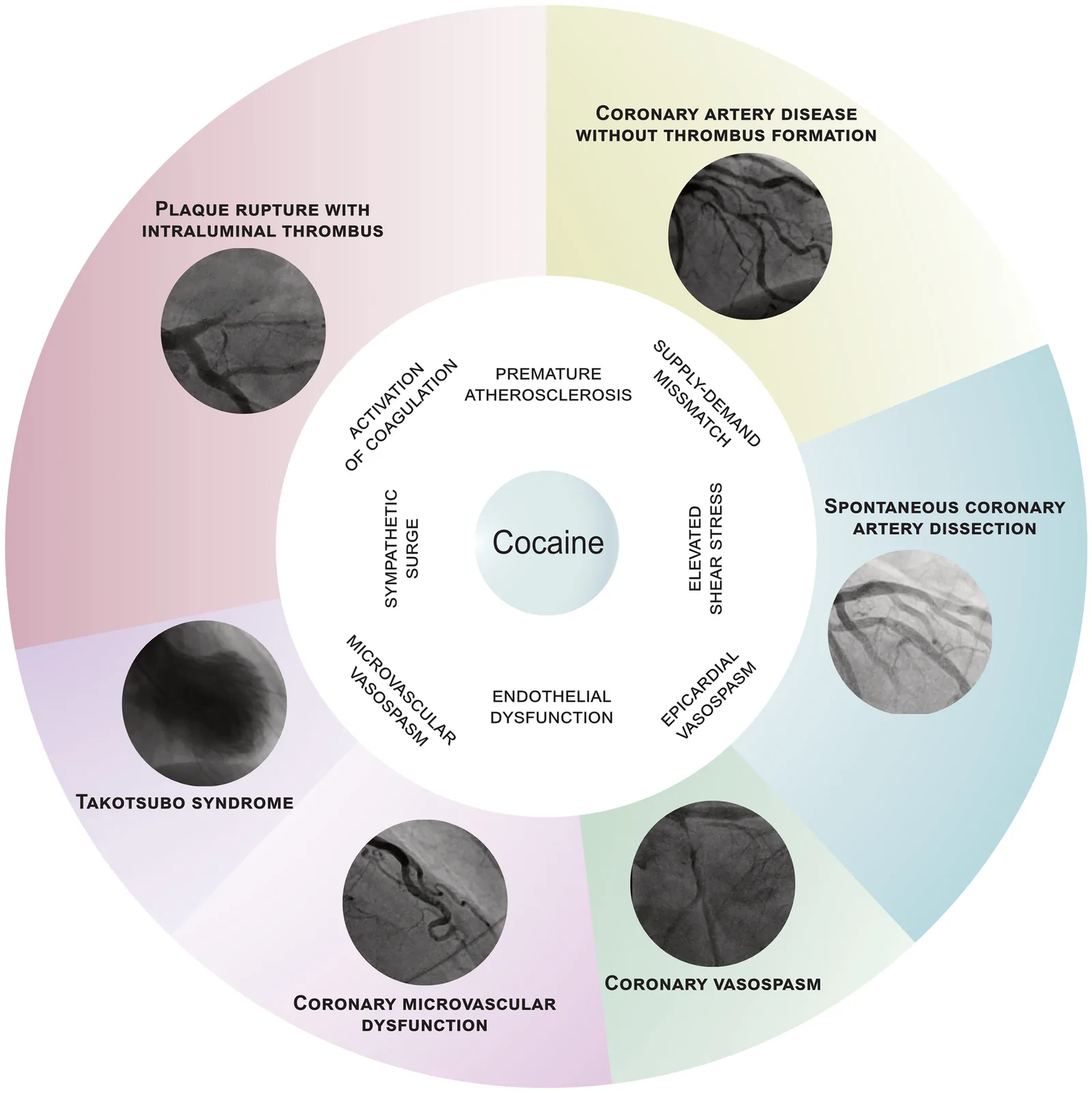

A broad variety of etiologies of acute myocardial infarction was found among patients with cocaine-induced acute myocardial infarction. The most common etiology was atherosclerotic coronary artery disease with or without intraluminal thrombus formation. However, slightly more than half of the patients presented with non-atherosclerotic causes of myocardial infarction. Among those, spontaneous coronary artery dissection and coronary vasospasm were found as epicardial diseases, and coronary microvascular dysfunction and Takotsubo syndrome as microvascular effects of cocaine consumption. This variety can be explained by the multifold pathophysiological mechanisms of cocaine. It is known as a risk factor for premature atherosclerosis and as a potent sympathomimetic, which leads to elevated blood pressure, inotropy, increased heart rate, and vasoconstriction. Increased shear stress might provoke plaque rupture and spontaneous coronary artery dissection. Furthermore, cocaine is known as a prothrombotic agent. Supply–demand mismatch and superimposed vasospasm are reasons for myocardial infarction in coronary artery disease without thrombus formation. Microvascular vasospasm and endothelial dysfunction might cause coronary microvascular dysfunction and Takotsubo syndrome after cocaine consumption.

## Introduction

The use of cocaine as a recreational drug has reached epidemic levels in the 1980s and has further increased over the recent years. It is the second most common drug consumed in Europe after cannabis. Cocaine accounts for most drug-related emergency department consultations and is associated with serious health issues [1]. The most frequent symptom is chest pain, and up to 9.7% of these patients are diagnosed with an acute myocardial infarction (AMI) [2, 3]. Cocaine-induced AMI (CI-AMI) was estimated to account for 25% of AMI among younger patients between 18 and 45 years of age [4].

The association between cocaine use and AMI was first reported in 1982 and subsequently documented in a number of case reports and clinical studies [5, 6]. The pathophysiological mechanisms leading to myocardial ischemia are multifactorial. Cocaine is a potent sympathomimetic drug by inhibition of the presynaptic reuptake of norepinephrine and dopamine. It increases the oxygen demand by increased afterload and positive chronotropic and inotropic effects, leads to coronary vasoconstriction, and may induce thrombus formation in the coronary arteries. Premature atherosclerotic coronary artery disease has also been observed in long-term cocaine abusers [6, 7].

Data on CI-AMI are still rare, and most clinical studies have been performed decades ago. Angiographic studies in this collective focused on the description of atherosclerotic coronary artery disease (CAD) [8–13]. However, a significant proportion of CI-AMI patients has non-obstructive coronary arteries, and the above-mentioned pathomechanisms indicate other important causes of AMI that need to be addressed in the management of these patients. The aim of this study was to define causative pathologies of CI-AMI and to analyze patient demographics, presentation, and outcomes.

## Methods

### Study population

Patients with the diagnosis of CI-AMI from January 1997 to December 2023 at the University Hospital Zurich, Switzerland, were eligible for inclusion in the study. Patients were retrospectively included after diagnosis of AMI during clinical routine. They were identified by data query of the electronic medical records for “myocardial infarction” and “cocaine”. Patients with self-reported cocaine consumption before AMI or positive urine test for cocaine were included. AMI was defined according to the fourth universal definition of myocardial infarction [14]. Angiograms were reviewed by two experienced interventional cardiologists to confirm the diagnosis. Plaque rupture was assumed, when a plaque with an intraluminal thrombus was identified. Angiographic criteria described by Saw et al. [15] were applied for the diagnosis of spontaneous coronary artery dissection (SCAD). Coronary vasospasm was diagnosed, if a focal or diffuse stenosis with reversibility after nitroglycerine application was recorded. Coronary slow-flow, defined as the delayed opacification of the coronary vasculature [16], in the absence of other pathologies was used as an angiographic surrogate for the diagnosis of coronary microvascular disease (CMD). Takotsubo syndrome (TTS) was diagnosed and classified as proposed by Templin et al. [17] Patients with unstable angina, chronic coronary syndromes, or denied consent were excluded from the study. The prevalence of CI-AMI was calculated by correlation with all cases of the acute coronary syndrome (ACS) database of the University Heart Center Zurich for the period from 2007 to 2023, in which ACS data was available.

### Assessment of coronary microvascular dysfunction

CMD was evaluated by retrospective, angiography-based analysis of all available coronary angiograms. Indices of angiographic microvascular resistance (AMR) were calculated by a single-view pressure-wire- and adenosine-free analysis using the AngioPlus Core software (Microport Medical Co., Shanghai, China). AMR values > 250 mmHg*s/m were used as a cut-off for CMD, which were demonstrated to correlate with an invasively measured index of microvascular resistance > 25 [18].

### Management and follow-up

Patients with CI-AMI were managed according to the judgement of the treating physicians. Hospital records were analyzed, and patient history, presentation, diagnostic results, treatment, in-hospital course, and major adverse cardiovascular events (MACE) during the first 30 days, 1 year, and 2 years were recorded. MACE were defined as the composite of cardiovascular death, myocardial infarction, unplanned revascularization, or stroke.

### Statistical analysis

Statistical analyses and compilation of graphs were performed using SPSS version 29.0 (IBM) and Prism version 10.2.0 (GraphPad). Continuous variables were provided as medians and interquartile ranges, and categorical variables as numbers with percentages. A p-value < 0.05 was considered statistically significant. Continuous variables were compared by the Mann–Whitney U test for two independent groups and by the Kruskal–Wallis test for more than two independent groups. Categorical variables were compared by the Pearson *χ*^2^ test or Fisher’s exact test. Kaplan Meier curves were generated for survival analyses and the log rank test was used for comparisons among groups.

### Ethics approval

The study protocol conforms to the ethical guidelines of the 1975 Declaration of Helsinki and was approved by the Cantonal Ethics Committee Zurich, Switzerland.

## Results

### Patient demographics and risk factors

Forty-five patients with CI-AMI were included in the study. Baseline characteristics are displayed in Table 1. CI-AMI accounted for 0.5% of ACS cases treated at the study center during the observation period. Three patients had recurrent AMI along with continued cocaine consumption. Most cases of CI-AMI occurred in male patients (n = 37, 82%) and the median age was 46 years (IQR, 37–52 years). The majority was low-educated (n = 24, 53%), single (n = 11, 24%) and had no children (n = 16, 36%). Most patients (n = 34, 76%) reported non-sporadic cocaine consumption, i.e. more than once per month, were multiple drug users (n = 31, 69%), and current or former smokers (n = 38, 80%). The prevalence of other cardiovascular risk factors was low (Table 1). A significant proportion of patients had psychiatric (n = 11, 25%), neurologic (n = 8, 18%), or chronic infective diseases (n = 8, 18%).

**Table 1:** Patient characteristics, presentation, and non-invasive diagnostics of patients with cocaine-induced acute myocardial infarction with and without coronary artery disease [N (%)]

	AMI associated with CAD	AMI not associated with CAD	All	*P* value*
Patients	20 (44)	25 (56)	45 (100)	
Age [median (IQR)]	47 (44–50)	44 (33–58)	46 (37–52)	0.33
Sex				
Male	18 (90)	19 (76)	37 (82)	0.22
Female	2 (10)	6 (24)	8 (18)	0.22
Non-sporadic cocaine consumption	16 (80)	18 (72)	34 (76)	0.54
Multiple drug abuse	13 (65)	18 (72)	31 (69)	0.61
Cardiovascular risk factors				
Hypertension	7 (35)	7 (28)	14 (31)	0.61
Diabetes mellitus	1 (5)	1 (4)	2 (4)	1.00
Smoking (active or former)	19 (95)	19 (76)	38 (84)	0.11
Dyslipidemia	7 (35)	3 (12)	10 (22)	0.08
Family history	3 (15)	4 (16)	7 (16)	1.00
Obesity	3 (15)	3 (12)	6 (13)	1.00
Comorbidities				
Psychiatric comorbidities	7 (35)	4 (16)	11 (24)	0.18
Neurologic comorbidities	3 (15)	5 (20)	8 (18)	0.72
Infective diseases	5 (25)	3 (12)	8 (18)	0.44
Clinical presentation				
Chest pain	17 (85)	19 (76)	36 (80)	0.71
Shortness of breath	0 (0)	6 (24)	6 (13)	0.03
Syncope	1 (5)	2 (8)	3 (7)	1.00
Cardiogenic shock	2 (10)	6 (24)	8 (18)	0.27
Pulmonary edema	0 (0)	1 (4)	1 (2)	1.00
Cardiac arrest	3 (15)	4 (16)	7 (16)	1.00
VT/VF	3 (15)	4 (16)	7 (16)	1.00
Hypertensive emergency	4 (21)	6 (24)	10 (22)	1.00
ACS type				
STEMI	14 (70)	11 (44)	25 (56)	0.08
NSTEMI	6 (30)	14 (56)	20 (44)	0.08
ECG				
ST segment elevation	13 (65)	10 (40)	23 (51)	0.10
ST segment depression	13 (68)	10 (40)	23 (51)	0.08
T wave inversion	11 (58)	8 (32)	19 (42)	0.09
LBBB	1 (5)	0 (0)	1 (2)	0.43
RBBB	1 (5)	3 (12)	4 (9)	0.62
QT prolongation	5 (26)	14 (56)	19 (42)	0.04
Lab values on admission				
Troponin [× ULN] [median (IQR)]	11.2 (1.3–20.1)	10.9 (2.3–38.6)	11.1 (2.1–28.4)	0.28
CK [× ULN] [median (IQR)]	0.8 (0.5–1.3)	1.6 (1.0–3.1)	1.2 (0.6–2.9)	0.01
NT-proBNP [× ULN] [median (IQR)]	3.7 (0.8–17.3)	3.5 (0.8–9.2)	3.5 (0.8–9.2)	0.88
Echocardiography				
LVEF [%] [median (IQR)]	51 (34–58)	51 (35–58)	51 (34–58)	0.98
Wall motion abnormalities	15 (79)	18 (72)	33 (73)	0.73

ACS, acute coronary syndrome; AMI, acute myocardial infarction; CAD, coronary artery disease; CK, creatine kinase; ECG, electrocardiogram; IQR, interquartile range; LBBB, left bundle branch block; LVEF, left ventricular ejection fraction; NSTEMI, non-ST-elevation myocardial infarction; NT-proBNP, N-terminal prohormone of brain natriuretic peptide; RBBB, right bundle branch block; STEMI, ST-elevation myocardial infarction; ULN, upper limit of normal; VF, ventricular fibrillation; VT, ventricular tachycardia

*P values calculated using Chi-square or Fisher's exact test for numbers and Kruskal Wallis Test for medians

### Patient presentation and non-invasive diagnostics

Most patients (n = 25, 56%) presented with ST-segment elevation myocardial infarction (STEMI), and the majority (n = 35, 78%) was diagnosed within 24 h after symptom onset. Chest pain was the principal symptom in 36 patients (80%), shortness of breath in 6 patients (13%), and syncope in 3 patients (7%). Seven patients (16%) were admitted after cardiac arrest due to ventricular fibrillation or ventricular tachycardia, 8 (18%) with cardiogenic shock, and 1 (2%) with pulmonary edema. Ten patients (22%) presented with a hypertensive emergency. ECG parameters, lab values, and echocardiography results are shown in Table 1.

### Coronary findings and angiographic characteristics

**Fig. 1:**
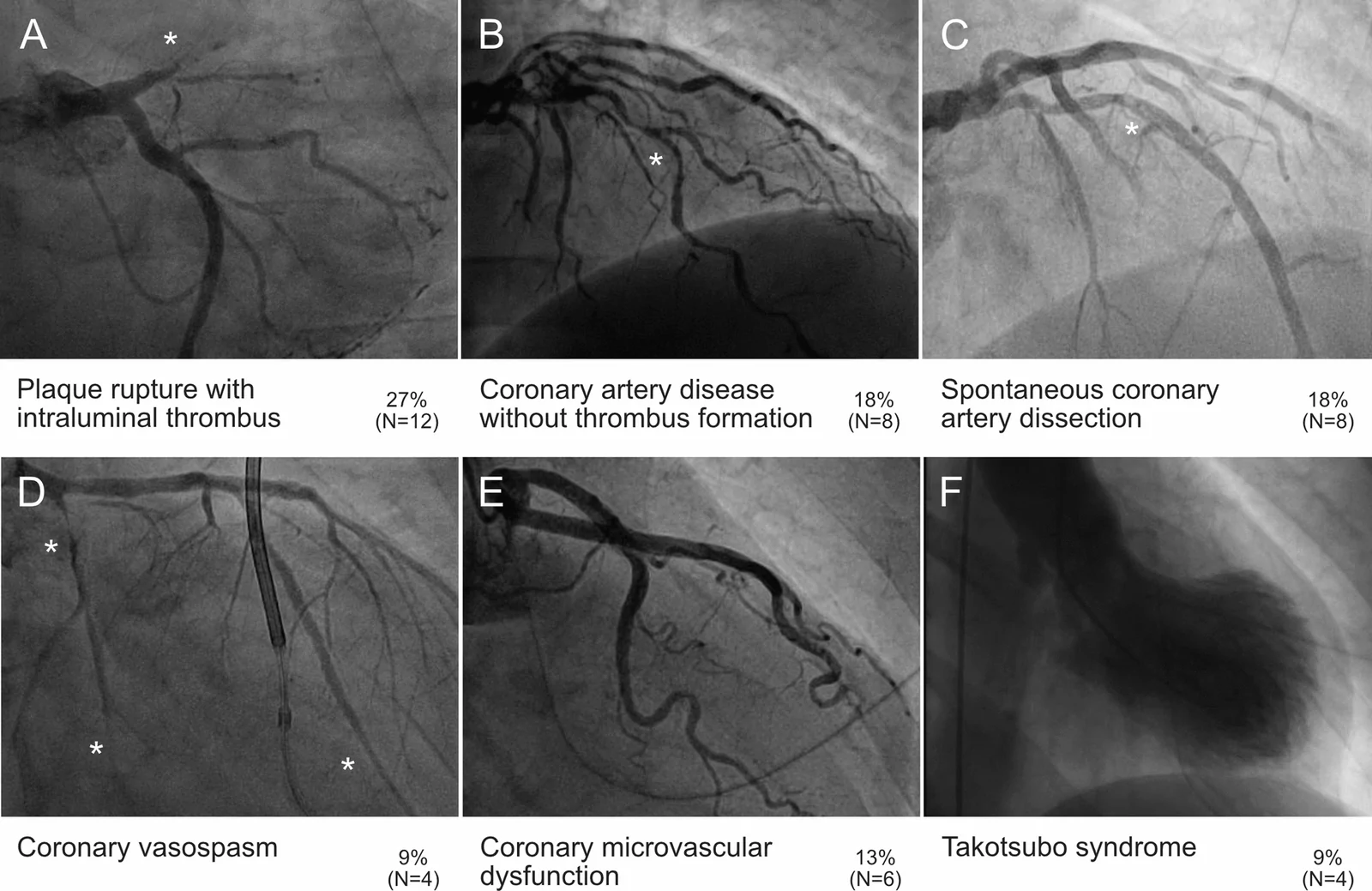
Etiologies of cocaine-induced acute myocardial infarction. The most frequent etiology of AMI was obstructive coronary artery disease with an intraluminal thrombus (**A**) or in the absence of thrombus formation (**B**). Non-atherosclerotic causes were found in slightly more than half of the patients and could be classified into spontaneous coronary artery dissection (**C**), coronary epicardial vasospasm (**D**), coronary microvascular dysfunction (**E**), and Takotsubo syndrome (**F**). Epicardial lesions are marked (*). No definitive diagnosis was found in 6% of patients (not included)

**Fig. 2:**
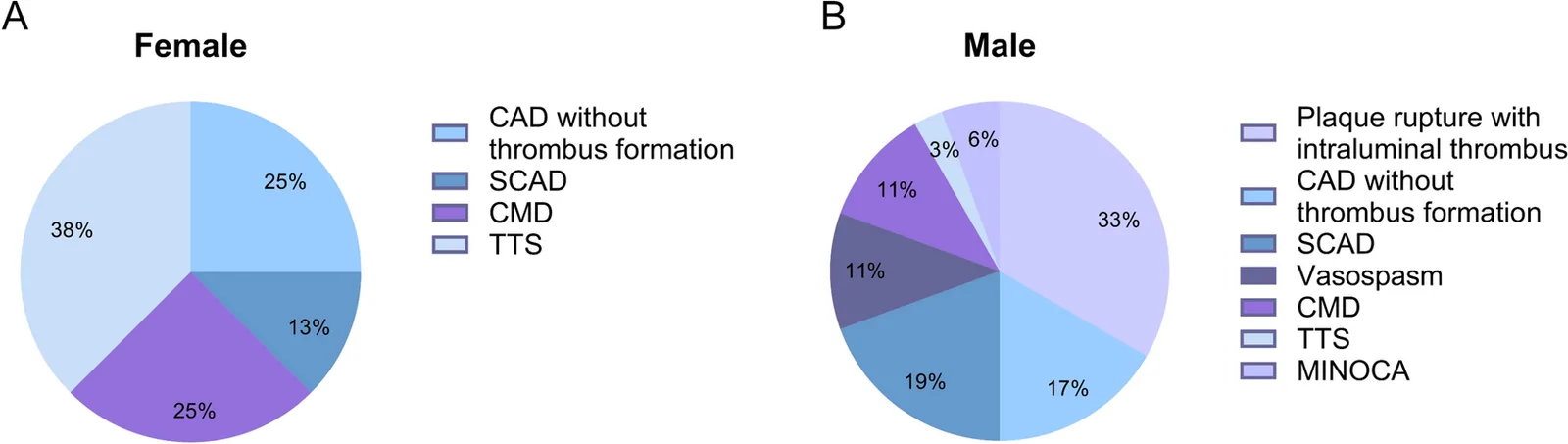
Sex differences in the distribution of etiologies of cocaine-induced acute myocardial infarction. Female patients were most often diagnosed with Takotsubo syndrome and coronary microvascular dysfunction and did not present with plaque rupture and intraluminal thrombus (**A**). Main diagnoses in male patients were coronary artery disease, spontaneous coronary artery dissection, and coronary epicardial vasospasm (**B**). Abbreviations: CAD, coronary artery disease; CMD, coronary microvascular dysfunction; MINOCA, myocardial infarction with non-obstructive coronary arteries; SCAD, spontaneous coronary artery disease; TTS, Takotsubo syndrome

Coronary angiography was performed in 42 patients. Invasive diagnostics were omitted in the remaining 3 patients after exclusion of obstructive CAD by noninvasive assessment. In 20 patients (44%), CAD was found as the underlying cause of AMI. Of those, 12 patients (27%) had plaque rupture with an intraluminal thrombus and 8 (18%) had CAD without thrombus formation. There was a broad variety of non-atherosclerotic causes of AMI in the remaining patients. SCAD was diagnosed in 8 (18%), coronary vasospasm in 4 (9%), and CMD in 6 patients (13%). TTS was invasively diagnosed in 2 patients and non-invasively in 2 other patients (9%). The diagnosis of myocardial infarction with non-obstructive coronary arteries (MINOCA) without a final etiology remained in 2 patients after invasive and in 1 after non-invasive evaluation (6%). Angiographic characteristics of these etiologies are shown in Fig. 1, and their detailed patient characteristics in Table 2. There were only minor differences in demographics, presentation, and noninvasive diagnostics of patients with and without obstructive CAD (Table 1). Female and male patients demonstrated a different distribution of these entities, however, without reaching statistical significance (Fig. 2). Nitro was used routinely during coronary angiography (n = 37, 88.1%), if there were no contraindications. The application of nitro during coronary angiography did not affect the in-hospital outcomes.

**Table 2:** Characteristics, management, and outcomes of different entities of cocaine-induced acute myocardial infarction [N (%)]

	Plaque rupture with thrombus	CAD without thrombus	SCAD	Vasospasm	CMD	TTS	MINOCA with unknown etiology	*P* value*
Patients	12 (27)	8 (18)	8 (18)	4 (9)	6 (14)	4 (9)	2 (5)	
Age [median (IQR)]	46 (40–49)	49 (45–56)	39 (37–54)	38 (28–56)	36 (22–66)	46 (39–51)	74 (66–74)	0.24
Sex								
Male	12 (100)	6 (75)	7 (88)	4 (100)	4 (67)	1 (25)	2 (100)	0.02
Female	0 (0)	2 (25)	1 (12)	0 (0)	2 (33)	3 (75)	0 (0)	0.02
ACS type								
STEMI	10 (83)	4 (50)	6 (75)	0 (0)	2 (33)	2 (50)	1 (50)	0.06
NSTEMI	2 (17)	4 (50)	2 (25)	4 (100)	4 (67)	2 (50)	1 (50)	0.06
Clinical presentation								
Chest pain	10 (83)	7 (88)	8 (100)	3 (75)	6 (100)	1 (25)	1 (50)	0.03
Shortness of breath	0 (0)	0 (0)	1 (13)	1 (25)	1 (17)	2 (50)	1 (50)	0.04
Syncope	1 (8)	0 (0)	0 (0)	0 (0)	0 (0)	2 (50)	0 (0)	0.10
Cardiogenic shock	2 (17)	0 (0)	1 (13)	1 (25)	0 (0)	2 (50)	1 (50)	0.16
Pulmonary edema	0 (0)	0 (0)	0 (0)	1 (25)	0 (0)	0 (0)	0 (0)	0.23
Cardiac arrest	3 (25)	0 (0)	2 (25)	0 (0)	0 (0)	0 (0)	1 (50)	0.27
VT/VF	3 (25)	0 (0)	2 (25)	0 (0)	0 (0)	0 (0)	1 (50)	0.27
Hypertensive emergency	2 (18)	2 (25)	1 (13)	1 (25)	3 (50)	0 (0)	1 (50)	0.53
Coronary artery disease								
Coronary 1-vessel disease	9 (75)	5 (63)						0.46
Coronary 2-vessel disease	2 (17)	0 (0)						0.35
Coronary 3-vessel disease	1 (8)	3 (38)						0.15
SCAD types								
Type 1			7 (88)					
Type 2			1 (12)					
Type 3			0 (0)					
TTS types								
Apical type						1 (25)		
Midventricular type						3 (75)		
Basal type						0 (0)		
Focal type						0 (0)		
Coronary tortuosity	2 (17)	0 (0)	2 (25)	0 (0)	2 (33)	1 (25)	1 (50)	0.05
Microvascular function								
AMR [mmHg*s/m] [median (IQR)]	319 (259–359)	238 (215–296)	295 (244–368)	297 (230–297)	356 (277–390)	319 (285–319)	310 (204–310)	0.52
CMD	9 (90)	5 (71)	6 (86)	2 (67)	6 (100)	2 (100)	1 (50)	0.48
Management								
Conservative	0 (0)	2 (25)	4 (50)	4 (100)	6 (100)	4 (100)	2 (100)	< 0.001
Revascularization	12 (100)	6 (75)	4 (50)	0 (0)	0 (0)	0 (0)	0 (0)	< 0.001
PCI	12 (100)	5 (63)	4 (50)	0 (0)	0 (0)	0 (0)	0 (0)	< 0.001
CABG	1 (8)	0 (0)	0 (0)	0 (0)	0 (0)	0 (0)	0 (0)	1.00
30-day outcome								
Acute heart failure and ventricular arrhythmias	3 (25)	1 (13)	1 (13)	1 (25)	1 (17)	2 (50)	1 (50)	0.72
Cardiogenic shock	2 (17)	1 (13)	1 (13)	1 (25)	0 (0)	2 (50)	1 (50)	0.38
Pulmonary edema	0 (0)	0 (0)	0 (0)	0 (0)	1 (17)	0 (0)	0 (0)	0.36
VT/VF	1 (8)	0 (0)	0 (0)	0 (0)	0 (0)	1 (25)	0 (0)	0.58
30-day MACE	2 (17)	3 (38)	1 (13)	0 (0)	0 (0)	0 (0)	1 (50)	0.35
Cardiovascular death	2 (17)	0 (0)	1 (13)	0 (0)	0 (0)	0 (0)	1 (50)	0.40
Myocardial infarction or unplanned revascularization	0 (0)	1 (13)	0 (0)	0 (0)	0 (0)	0 (0)	0 (0)	0.73
Stroke	0 (0)	2 (25)	0 (0)	0 (0)	0 (0)	0 (0)	0 (0)	0.25
1-year MACE	3 (25)	3 (43)	2 (33)	1 (50)	0 (0)	0 (0)	1 (50)	0.60
Cardiovascular death	2 (17)	0 (0)	1 (17)	1 (50)	0 (0)	0 (0)	1 (50)	0.35
Myocardial infarction or unplanned revascularization	0 (0)	1 (14)	1 (17)	0 (0)	0 (0)	0 (0)	0 (0)	0.71
Stroke	1 (8)	2 (29)	0 (0)	0 (0)	0 (0)	0 (0)	0 (0)	0.51
2-year MACE	3 (27)	3 (43)	2 (33)	1 (100)	0 (0)	0 (0)	2 (100)	0.18
Cardiovascular death	2 (20)	1 (14)	1 (20)	1 (100)	0 (0)	0 (0)	2 (100)	0.07
Myocardial infarction or unplanned revascularization	0 (0)	1 (14)	1 (17)	0 (0)	0 (0)	0 (0)	0 (0)	0.81
Stroke	1 (9)	2 (29)	0 (0)	0 (0)	0 (0)	0 (0)	0 (0)	0.64

ACS, acute coronary syndrome; AMR, angiographic microvascular resistance; CABG, coronary artery bypass graft; CAD, coronary artery disease; CMD, coronary microvascular dysfunction; IQR, interquartile range; MACE, major adverse cardiovascular events; MINOCA, myocardial infarction with non-obstructive coronary arteries; NSTEMI, non-ST-elevation myocardial infarction; PCI, percutaneous coronary intervention; SCAD, spontaneous coronary artery disease; STEMI, ST-elevation myocardial infarction; TTS, Takotsubo syndrome; ULN, upper limit of normal; VF, ventricular fibrillation; VT, ventricular tachycardia

*P values calculated using Chi-square test or Fisher's exact test for numbers and Kruskal Wallis Test for medians

Obstructive CAD was the underlying mechanism in most patients with STEMI (14, 56%) and in 30% (n = 6) of patients with non-ST-segment elevation myocardial infarction (NSTEMI). The majority was diagnosed with single vessel disease (n = 14, 70%), and most lesions (n = 12, 60%) were detected in the left anterior descending artery (LAD). Most patients without atherosclerotic CAD were diagnosed with SCAD (n = 8). The dissection most frequently involved the LAD (n = 6, 75%). Multivessel SCAD was found in 3 patients (38%). SCAD type 1 was the predominant type (n = 7, 88%). Tortuosity of the coronary arteries was found in 2 of the SCAD patients (25%). Coronary vasospasm was found in 4 patients. Vasospasm was diffuse in 1 case (25%) and focal in the remaining 3 patients (75%). Six patients without other coronary pathologies demonstrated coronary slow-flow as a correlate for CMD. TTS was found in 4 patients. The majority was diagnosed with the midventricular type (n = 3, 75%). The apical form of TTS was found in the remaining patient. These patients were predominantly female, only one reported chest pain, and all were diagnosed with QT prolongation. The diagnosis of MINOCA without a final etiology remained in 2 male patients after coronary angiography. One patient was diagnosed with coronary ectasia, and elevated AMR values were measured. The other patient presented with STEMI and was diagnosed with a chronic total occlusion of the right coronary artery (RCA), but no culprit lesion. Therefore, superimposed vasospasm was assumed as the reason of AMI.

### Assessment of coronary microvascular dysfunction

Coronary angiograms were available for assessment of CMD in 42 cases. AMR values were analyzed in 80 vessels of 37 patients after exclusion of hemodynamically relevant stenoses, which precluded an analysis of AMR. Median AMR values were 303 mmHg*s/m (IQR, 257–352 mmHg*s/m) in the LAD, 331 mmHg*s/m (IQR, 264–406 mmHg*s/m) in the left circumflex artery (LCX), 289 mmHg*s/m (IQR, 239–369 mmHg*s/m) in the RCA, and 368 mmHg*s/m (IQR, 241–368 mmHg*s/m) on average over all coronary vessels. AMR values consistent with CMD in at least one coronary vessel were found in 31 patients (91%), hereof 18 (82%) in the LAD, 25 (78%) in the LCX, and 18 (72%) in the RCA. The proportion of patients with CMD of non-stenotic vessels were not significantly different in patients with and without CAD and in the different etiologies of coronary lesions (Table 2).

### Management and outcomes

Half of the patients (n = 22, 49%) were treated by coronary revascularization (Table 2). Percutaneous coronary intervention (PCI) was performed in 21 of these patients (47%). The remaining patient received a coronary artery bypass graft (CABG). Of those, 18 patients (82%) had CAD, and 4 cases (18%) were diagnosed with SCAD.

Median follow-up of patients was 24 months (IQR, 6–51 months). The in-hospital course and 30-day outcomes were characterized by a significant number of adverse cardiovascular events (Table 2). MACE occurred in 7 patients (16%) during the first 30 days after diagnosis. Four patients (9%) died due to a cardiovascular cause, 2 patients (4%) developed stroke, and 1 patient (2%) needed an unplanned revascularization. Furthermore, 1 patient (2%) each developed cardiogenic shock and pulmonary edema, and 3 patients (7%) developed ventricular arrhythmias during the hospital course.

Long-term follow-up was available in 36 patients (80%) after 1 year and in 30 patients (67%) after 2 years. MACE were documented in 28% after 1 year and in 34% after 2 years. Cardiovascular death occurred in 14% and in 23%, stroke in 8% and 10%, and myocardial infarction or unplanned revascularization in 6% and 7% after 1 and 2 years, respectively (Table 2).

Rates of cardiogenic shock, tachyarrhythmias, stroke, cardiovascular death, and overall MACE were comparable between AMI types, entities of CI-AMI, and patients with normal or elevated AMR.

## Discussion

This study demonstrates a prevalence of CI-AMI of about 0.5% in patients undergoing coronary angiography for AMI. It is the first study that classifies the various angiographic entities of CI-AMI and describes their patient characteristics, management, and short-term outcomes. Almost half of the patients presented with CAD. In addition, a broad range of entities was found beside CAD, specifically SCAD in 18%, CMD in 13%, coronary vasospasm in 9%, and TTS in 9%. Revascularization was performed in about half of the patients due to CAD or high-risk SCAD. Patients with CI-AMI frequently had an unfavorable short-term outcome with 16% MACE and almost 10% cardiovascular deaths.

### Patient characteristics, presentation, and prediction of coronary artery disease

Most patients had a first event of AMI, even if they were non-sporadic cocaine consumers. Patients predominantly were younger than 50 years, had no established family environment and a low social status. The majority reported non-sporadic cocaine consumption and multiple drug abuse. These findings are consistent with other studies [3, 19]. Most patients were men and smokers. However, the prevalence of other classic cardiovascular risk factors was low in this patient cohort. These results support other studies that demonstrated a younger age and fewer cardiovascular risk factors in CI-AMI patients than in ACS patients in general [7, 20, 21]. These circumstances lead to a poor prognostic value of established risk stratifying scores for ACS [22, 23]. More than half of patients presented with STEMI and the majority of patients had repolarization abnormalities in the initial ECG. A significant proportion of patients presented with a hypertensive emergency, however, the proportion of these patients among patients with the final diagnosis of CAD and non-atherosclerotic AMI was not different. CAD patients had some significant differences in their presentation and noninvasive diagnostics compared with AMI patients without the final diagnosis of CAD: they had a higher BMI, less often shortness of breath as the principal symptom, more frequently repolarization abnormalities and less frequently QT prolongation in the initial ECG, and a lower CK elevation at presentation. However, these parameters were no clinically useful discriminators of CAD, since many patients without CAD demonstrated comparable patient characteristics. Other than previously described [9], age and the number of classic cardiovascular risk factors were not significantly different between patients with and without CAD in this study. In summary, we did not identify reliable clinical predictors for CAD in CI-AMI. Therefore, we propose an invasive approach for the management of CI-AMI, which has been demonstrated to improve outcomes in these patients [24].

### Angiographic characteristics, pathophysiology of cocaine-induced acute myocardial infarction, and the role of coronary microvascular dysfunction

Patients with CI-AMI were diagnosed with CAD in almost half of the cases, most of them with an initial diagnosis of a coronary single vessel disease. The number of patients with CAD was in the range of previously published cohorts (36–82%) [9–11, 13, 25]. Cocaine consumption seems to be a risk factor for premature CAD, since patients were younger and had, besides smoking, few cardiovascular risk factors. Another study reported a significantly higher plaque burden as analyzed by coronary computed tomography angiography in cocaine consumers with chest pain compared with matched patients without cocaine-consumption [26]. The mechanisms of cocaine leading to AMI, beyond premature CAD, are multifold. Cocaine is a potent sympathomimetic and leads to an increased heart rate, contractility, blood pressure, and thus, myocardial oxygen demand. It causes vasoconstriction by the stimulation of α-adrenergic receptors on smooth muscles of the coronary arteries, increased levels of endothelin, and reduced levels of nitric oxide. Furthermore, cocaine induces a prothrombotic state by a variety of mechanisms [7]. In summary, these pathophysiologic changes lead to plaque rupture and coronary thrombus formation in a significant proportion of patients with CAD.

These pathomechanisms can also cause AMI in the absence of CAD. More than half of patients in this study presented with AMI without CAD. Only 9% were diagnosed with coronary vasospasm during coronary angiography, a mechanism that is often assumed by physicians in these patients. This may be a reason for the demonstrated safety of beta blockers in patients with cocaine-induced chest pain [27]. Vasospasm and elevated blood pressure might result in an increased shear stress in the coronary arteries, which is assumed a key factor in the development of SCAD [28, 29]. Eighteen percent of patients with CI-AMI in this patient cohort were diagnosed with SCAD, a much higher proportion than in patients with ACS in general [30]. Interestingly, almost all patients were male, there was a high rate of SCAD type 1 and multivessel SCAD, and few patients demonstrated tortuous coronary vessels. In contrast, published cohorts of SCAD patients are predominantly female with SCAD type 2 being the most frequent angiographic type, and there is a high prevalence of coronary tortuosity [30, 31]. These differences might indicate different pathophysiological mechanisms in cocaine-induced SCAD. Increased shear stress might be more important than other established risk factors, such as hormonal changes or vessel wall alterations like fibromuscular dysplasia in these patients.

In addition to these epicardial mechanisms of AMI, we identified a central role of CMD in patients with CI-AMI. Even if only 9% of patients were diagnosed with coronary slow-flow during coronary angiography, increased microvascular resistance was found in the majority of patients by analysis of AMR. These findings were present both in patients with and without CAD. Acute coronary microvascular spasm (MVS) can be postulated to be the underlying cause of AMI at least in a proportion of patients, which is an established differential diagnosis in patients with MINOCA [32]. In the remaining patients, elevated microvascular resistance might be a bystander, either due to acute MVS or because of preexistent CMD. The rate of preexistent CMD in patients with non-sporadic cocaine consumption is unclear and needs to be elucidated in further studies. Nine percent of patients in the current study were diagnosed with TTS. TTS is an acute and transient heart failure syndrome following emotional or physical triggers. CMD was detected in all patients with TTS in another study [33] and was postulated to be a key mechanism in the pathophysiology of TTS [34]. Therefore, it could be assumed that acute MVS is one link between cocaine consumption and TTS. Interestingly, most patients with TTS in this study presented with the midventricular type, which is known to appear most frequently after physical triggers and in young and male patients [34].

In summary, a variety of underlying pathologies can be identified in CI-AMI. The most important is CAD with or without intraluminal thrombus. However, non-atherosclerotic causes, which are usually rare in ACS cohorts [32, 35], are diagnosed in a significant proportion of patients presenting with CI-AMI and need to be considered in the differential diagnosis of these patients.

### Management and outcomes

The number of patients with CI-AMI in need of revascularization was high. Around half of the patients were treated by PCI or CABG, which was the consequence of a high rate of CAD. A smaller proportion of patients were treated with revascularization due to high-risk SCAD. These findings strongly support an invasive approach, and routine coronary angiography should be recommended in all patients with CI-AMI. Conservative management has been shown to be associated with worse outcomes in CI-AMI compared to an invasive management [24].

Patients with CI-AMI should be considered as high-risk patients, since unfavorable outcomes can frequently be observed. Patient were frequently admitted after cardiac arrest, and cardiogenic shock or pulmonary edema was present even in one fifth of patients. The 30-day MACE rate was high, and almost ten percent of patients died within this period. An increasing number of adverse events also marked the follow-up episode over 2 years. Rates of MACE and cardiovascular death of CI-AMI patients in this study were substantially higher than those of general ACS patients published in national and international ACS cohorts (MACE: 16% vs. 1.2–9.5% at 30 days and 28% vs. 7.3–9.5% at 1 year; cardiovascular death: 9% vs. 0.2–1.8%% at 30 days and 14% vs. 1.4–2.8% at 1 year) [36–40]. These findings are comparable to another study on outcomes in CI-AMI, which found significantly higher MACE and cardiovascular mortality rates among cocaine-induced ACS compared with ACS not associated with cocaine consumption [20]. Of note, the risk of adverse events was independent of the presence of CAD, the type of AMI, and the definite diagnosis. We therefore recommend a prolonged observation of patients independent of the findings of coronary angiography.

### Limitations of the study

Several limitations must be considered for this study. First, it is important to mention the retrospective nature of the analysis. Patients with CI-AMI were identified during clinical management. Urine tests for cocaine were not performed routinely and most patients were included after self-reported cocaine consumption before AMI. Therefore, the rate of CI-AMI might be underestimated. The frequency and duration of cocaine use was not analyzed systematically, and thus, its impact on adverse events cannot be evaluated. Second, the study was performed in a monocentric manner in a tertiary referral-center. Results might not be applicable to all local conditions of cardiovascular care. Despite a prolonged observation period, the number of cases was rather low. Therefore, the significance of differences in patient characteristics and outcome data is limited in this study, especially when looking at the identified etiologies of AMI. Thus, adequately powered prospective and multicentric studies are needed to assess these aspects.

## Conclusions

CI-AMI is a rare, but important cause of ACS. CAD is the most frequent etiology of AMI in this patient group, which indicates an invasive approach with a consecutive revascularization in a significant proportion of patients. Therefore, routine coronary angiography is strongly recommended in all patients with CI-AMI. However, there is a broad range of entities leading to AMI beside CAD. Vasospasm, SCAD, CMD, and TTS were found in about half of the patients in this study collective. Patients with CI-AMI may need to be considered as high-risk patients, and the number of adverse events is significant.

## Data Availability

Data generated or analyzed during the study are available from the corresponding author by request.

## Abbreviations

ACS: Acute coronary syndrome
AMI: Acute myocardial infarction
AMR: Angiographic microvascular resistance
CI-AMI: Cocaine-induced acute myocardial infarction
CABG: Coronary artery bypass graft
CAD: Coronary artery disease
CK: Creatine kinase
CMD: Coronary microvascular disease
ECG: Electrocardiogram
HIV: Human immunodeficiency virus
LAD: Left anterior descending artery
LCX: Left circumflex artery
LVEF: Left ventricular ejection fraction
MACE: Major adverse cardiovascular events
MINOCA: Myocardial infarction with non-obstructive coronary arteries
MVS: Microvascular spasm
NSTEMI: Non-ST-elevation myocardial infarction
NT-proBNP: N-terminal prohormone of brain natriuretic peptide
PCI: Percutaneous coronary intervention
RCA: Right coronary artery
SCAD: Spontaneous coronary artery dissection
STEMI: ST-elevation myocardial infarction
TTS: Takotsubo syndrome
ULN: Upper limit of normal

## References

[1] European Monitoring Centre for Drugs and Drug Addiction. Cocaine – the current situation in Europe (European Drug Report 2023). 16 June 2023; available from: https://www.emcdda.europa.eu/publications/european-drug-report/2023/cocaine_en.

[2] Weber JE, Chudnofsky CR, Boczar M, Boyer EW, Wilkerson MD, Hollander JE (2000) Cocaine-associated chest pain: how common is myocardial infarction? Acad Emerg Med 7(8):873–877 10958126 10.1111/j.1553-2712.2000.tb02064.x

[3] Sami F, Chan WC, Acharya P, Sethi P, Cannon C, Hockstad ES et al (2022) Outcomes in patients with history of cocaine use presenting with chest pain to the emergency department: Insights from the Nationwide Emergency Department Sample 2016–2018. J Am Coll Emerg Physicians Open 3(1):e12618 35072159 10.1002/emp2.12618 PMC8760951

[4] Qureshi AI, Suri MF, Guterman LR, Hopkins LN (2001) Cocaine use and the likelihood of nonfatal myocardial infarction and stroke: data from the Third National Health and Nutrition Examination Survey. Circulation 103(4):502–506 11157713 10.1161/01.cir.103.4.502

[5] Coleman DL, Ross TF, Naughton JL (1982) Myocardial ischemia and infarction related to recreational cocaine use. West J Med 136(5):444–446 7101910 PMC1273814

[6] Lange RA, Hillis LD (2001) Cardiovascular complications of cocaine use. N Engl J Med 345(5):351–358 11484693 10.1056/NEJM200108023450507

[7] McCord J, Jneid H, Hollander JE, de Lemos JA, Cercek B, Hsue P et al (2008) Management of cocaine-associated chest pain and myocardial infarction: a scientific statement from the American Heart Association Acute Cardiac Care Committee of the Council on Clinical Cardiology. Circulation 117(14):1897–1907 18347214 10.1161/CIRCULATIONAHA.107.188950

[8] Hollander JE, Hoffman RS, Burstein JL, Shih RD, Thode HC Jr (1995) Cocaine-associated myocardial infarction. Mortality and complications. Cocaine-Associated Myocardial Infarction Study Group. Arch Intern Med 155(10):1081–1086 7748052

[9] Hollander JE, Shih RD, Hoffman RS, Harchelroad FP, Phillips S, Brent J et al (1997) Predictors of coronary artery disease in patients with cocaine-associated myocardial infarction. Cocaine-Associated Myocardial Infarction (CAMI) Study Group. Am J Med 102(2):158–163 9217565 10.1016/s0002-9343(96)00406-8

[10] Kontos MC, Jesse RL, Tatum JL, Ornato JP (2003) Coronary angiographic findings in patients with cocaine-associated chest pain. J Emerg Med 24(1):9–13 12554033 10.1016/s0736-4679(02)00660-1

[11] Mohamad T, Niraj A, Farah J, Obideen M, Badheka A, Kondur A et al (2009) Spectrum of electrocardiographic and angiographic coronary artery disease findings in patients with cocaine-associated myocardial infarction. Coron Artery Dis 20(5):332–336 19543086 10.1097/MCA.0b013e32832b906c

[12] Carrillo X, Curos A, Muga R, Serra J, Sanvisens A, Bayes-Genis A (2011) Acute coronary syndrome and cocaine use: 8-year prevalence and inhospital outcomes. Eur Heart J 32(10):1244–1250 21266375 10.1093/eurheartj/ehq504

[13] Sehatbakhsh S, Kushnir A, Furlan S, Donath E, Ghumman W, Chait R (2018) Importance of a risk stratification strategy to identify high-risk patients presenting with cocaine-associated acute coronary syndrome. Crit Pathw Cardiol 17(3):147–150 30044255 10.1097/HPC.0000000000000147

[14] Thygesen K, Alpert JS, Jaffe AS, Chaitman BR, Bax JJ, Morrow DA et al (2019) Fourth universal definition of myocardial infarction (2018). Eur Heart J 40(3):237–269 30165617 10.1093/eurheartj/ehy462

[15] Saw J (2014) Coronary angiogram classification of spontaneous coronary artery dissection. Catheter Cardiovasc Interv 84(7):1115–1122 24227590 10.1002/ccd.25293

[16] Aparicio A, Cuevas J, Moris C, Martin M (2022) Slow coronary blood flow: pathogenesis and clinical implications. Eur Cardiol 17:e08 35356630 10.15420/ecr.2021.46 PMC8941644

[17] Templin C, Ghadri JR, Diekmann J, Napp LC, Bataiosu DR, Jaguszewski M et al (2015) Clinical features and outcomes of Takotsubo (stress) cardiomyopathy. N Engl J Med 373(10):929–938 26332547 10.1056/NEJMoa1406761

[18] Fan Y, Fezzi S, Sun P, Ding N, Li X, Hu X et al (2022) In vivo validation of a novel computational approach to assess microcirculatory resistance based on a single angiographic view. J Pers Med 12(11):1798 36573725 10.3390/jpm12111798 PMC9692562

[19] Booth BM, Weber JE, Walton MA, Cunningham RM, Massey L, Thrush CR et al (2005) Characteristics of cocaine users presenting to an emergency department chest pain observation unit. Acad Emerg Med 12(4):329–337 15805324 10.1197/j.aem.2004.11.021

[20] Carrillo X, Vilalta V, Cediel G, Fernandez-Nofrerias E, Rodriguez-Leor O, Mauri J et al (2019) Trends in prevalence and outcomes of acute coronary syndrome associated with cocaine consumption: the RUTI-cocaine study. Int J Cardiol 283:23–27 30595359 10.1016/j.ijcard.2018.12.026

[21] Gupta N, Washam JB, Mountantonakis SE, Li S, Roe MT, de Lemos JA et al (2014) Characteristics, management, and outcomes of cocaine-positive patients with acute coronary syndrome (from the National Cardiovascular Data Registry). Am J Cardiol 113(5):749–756 24388623 10.1016/j.amjcard.2013.11.023

[22] Faramand Z, Martin-Gill C, Frisch SO, Callaway C, Al-Zaiti S (2021) The prognostic value of HEART score in patients with cocaine associated chest pain: an age-and-sex matched cohort study. Am J Emerg Med 45:303–308 33041125 10.1016/j.ajem.2020.08.074 PMC7910314

[23] Chase M, Brown AM, Robey JL, Zogby KE, Shofer FS, Chmielewski L et al (2007) Application of the TIMI risk score in ED patients with cocaine-associated chest pain. Am J Emerg Med 25(9):1015–1018 18022495 10.1016/j.ajem.2007.03.004

[24] Arora S, Jaswaney R, Jani C, Zuzek Z, Thakkar S, Patel M et al (2021) Invasive approaches in the management of cocaine-associated non-ST-segment elevation myocardial infarction. JACC Cardiovasc Interv 14(6):623–636 33736770 10.1016/j.jcin.2021.01.005

[25] Rodriguez-Esteban M, Mesa-Fumero J, Facenda-Lorenzo M, Dorta-Macias C, Ramos-Lopez M, Soriano-Vela E (2009) Acute coronary syndrome and cocaine. Med Clin (Barc) 133(4):132–134 19371914 10.1016/j.medcli.2008.10.054

[26] Ebersberger U, Sudarski S, Schoepf UJ, Bamberg F, Tricarico F, Apfaltrer P et al (2013) Atherosclerotic plaque burden in cocaine users with acute chest pain: analysis by coronary computed tomography angiography. Atherosclerosis 229(2):443–448 23880201 10.1016/j.atherosclerosis.2013.05.032

[27] Espana Schmidt C, Pastori L, Pekler G, Visco F, Mushiyev S (2015) Early use of beta blockers in patients with cocaine associated chest pain. Int J Cardiol Heart Vasc 8:167–169 28785697 10.1016/j.ijcha.2015.06.001 PMC5497278

[28] Candreva A, Lodi Rizzini M, Schweiger V, Gallo D, Montone RA, Würdinger M et al (2023) Is spontaneous coronary artery dissection (SCAD) related to local anatomy and hemodynamics? An exploratory study. Int J Cardiol 386:1–7 37201616 10.1016/j.ijcard.2023.05.006

[29] Adlam D, Alfonso F, Maas A, Vrints C, Writing C (2018) European Society of Cardiology, acute cardiovascular care association, SCAD study group: a position paper on spontaneous coronary artery dissection. Eur Heart J 39(36):3353–3368 29481627 10.1093/eurheartj/ehy080 PMC6148526

[30] Würdinger M, Schweiger V, Gilhofer T, Cammann VL, Badorff A, Koleva I et al (2024) Twenty-five-year trends in incidence, angiographic appearance, and management of spontaneous coronary artery dissection. Int J Cardiol 395:131429 37827283 10.1016/j.ijcard.2023.131429

[31] Saw J, Starovoytov A, Humphries K, Sheth T, So D, Minhas K et al (2019) Canadian spontaneous coronary artery dissection cohort study: in-hospital and 30-day outcomes. Eur Heart J 40(15):1188–1197 30698711 10.1093/eurheartj/ehz007 PMC6462308

[32] Agewall S, Beltrame JF, Reynolds HR, Niessner A, Rosano G, Caforio AL et al (2017) ESC working group position paper on myocardial infarction with non-obstructive coronary arteries. Eur Heart J 38(3):143–153 28158518 10.1093/eurheartj/ehw149

[33] Schweiger V, Gilhofer T, Fang R, Candreva A, Seifert B, Di Vece D et al (2023) Coronary microvascular dysfunction in Takotsubo syndrome: an analysis using angiography-derived index of microcirculatory resistance. Clin Res Cardiol 113:1629–1637 37985475 10.1007/s00392-023-02329-7 PMC11579140

[34] Ghadri JR, Wittstein IS, Prasad A, Sharkey S, Dote K, Akashi YJ et al (2018) International expert consensus document on Takotsubo Syndrome (part I): clinical characteristics, diagnostic criteria, and pathophysiology. Eur Heart J 39(22):2032–2046 29850871 10.1093/eurheartj/ehy076 PMC5991216

[35] Anderson JL, Morrow DA (2017) Acute myocardial infarction. N Engl J Med 376(21):2053–2064 28538121 10.1056/NEJMra1606915

[36] Vranckx P, Frigoli E, Rothenbuhler M, Tomassini F, Garducci S, Ando G et al (2017) Radial versus femoral access in patients with acute coronary syndromes with or without ST-segment elevation. Eur Heart J 38(14):1069–1080 28329389 10.1093/eurheartj/ehx048

[37] Landolff Q, Quillot M, Picard F, Henry P, Sideris G, Bizeau O et al (2023) In-hospital and 1-year clinical results from the French Registry using polymer-free sirolimus-eluting stents in acute coronary syndrome and stable coronary artery disease. J Interv Cardiol 2023:8907315 38125031 10.1155/2023/8907315 PMC10733033

[38] Schoenenberger AW, Radovanovic D, Stauffer JC, Windecker S, Urban P, Niedermaier G et al (2011) Acute coronary syndromes in young patients: presentation, treatment and outcome. Int J Cardiol 148(3):300–304 19942306 10.1016/j.ijcard.2009.11.009

[39] Obeid S, Frangieh AH, Raber L, Yousif N, Gilhofer T, Yamaji K et al (2018) Prognostic value of SYNTAX score II in patients with acute coronary syndromes referred for invasive management: a subanalysis from the SPUM and COMFORTABLE AMI cohorts. Cardiol Res Pract 2018:9762176 30356345 10.1155/2018/9762176 PMC6176297

[40] Stähli BE, Varbella F, Linke A, Schwarz B, Felix SB, Seiffert M et al (2023) Timing of complete revascularization with multivessel PCI for myocardial infarction. N Engl J Med 389(15):1368–1379 37634190 10.1056/NEJMoa2307823

